# The Resilience of Cardiac Care Through Virtualized Services During the COVID-19 Pandemic: Case Study of a Heart Function Clinic

**DOI:** 10.2196/25277

**Published:** 2021-05-05

**Authors:** Amika Shah, Milena Guessi, Sahr Wali, Patrick Ware, Michael McDonald, Mary O'Sullivan, Juan Duero Posada, Heather Ross, Emily Seto

**Affiliations:** 1 Centre for Global eHealth Innovation Techna Institute University Health Network Toronto, ON Canada; 2 Institute of Health Policy, Management and Evaluation Dalla Lana School of Public Health University of Toronto Toronto, ON Canada; 3 Department of Computer Systems Institute of Mathematics and Computer Science University of São Paulo São Paulo Brazil; 4 Peter Munk Cardiac Centre University Health Network Toronto, ON Canada; 5 Ted Rogers Centre for Heart Research University Health Network Toronto, ON Canada; 6 Advanced Heart Failure, Transplantation and Mechanical Circulatory Support Toronto Western Hospital University Health Network Toronto, ON Canada; 7 Department of Medicine University of Toronto Toronto, ON Canada

**Keywords:** telemedicine, telehealth, digital health, digital medicine, COVID-19, coronavirus, SARS-CoV-2, public health, surveillance, outbreak, pandemic, infectious disease, cardiology, patient, organizational innovation, organizational objectives, global health, resilience

## Abstract

**Background:**

Virtual care has historically faced barriers to widespread adoption. However, the COVID-19 pandemic has necessitated the rapid adoption and expansion of virtual care technologies. Although the intense and prolonged nature of the COVID-19 pandemic has renewed people’s interest in health systems resilience, which includes how services adapt or transform in response to shocks, evidence regarding the role of virtual care technologies in health systems resilience is scarce.

**Objective:**

At Toronto General Hospital in Ontario, Canada, the rapid virtualization of cardiac care began on March 9, 2020, as a response to the pandemic. The objective of this study was to understand people’s experiences with and the barriers and facilitators of the rapid virtualization and expansion of cardiac care resulting from the pandemic.

**Methods:**

A single-case study was conducted with 3 embedded units of analysis. Patients, clinicians, and staff were recruited purposively from an existing mobile, phone-based telemonitoring program at a heart function clinic in Toronto, Canada. Individual, semistructured phone interviews were conducted by two researchers and transcribed verbatim. An inductive thematic analysis at the semantic level was used to analyze transcripts and develop themes.

**Results:**

A total of 29 participants were interviewed, including patients (n=16), clinicians (n=9), and staff (n=4). The following five themes were identified: (1) patient safety as a catalyst for virtual care adoption; (2) piecemeal virtual care solutions; (3) confronting new roles and workloads; (4) missing pieces in virtual care; and (5) the inequity paradox. The motivation to protect patient safety and a piecemeal approach to virtual care adoption facilitated the absorptive and adaptive resilience of cardiac care during the COVID-19 pandemic. However, ad hoc changes to clinic roles and workflows, challenges in building relationships through remote methods, and widened inequities were barriers that threatened virtual care sustainment.

**Conclusions:**

We contend that sustaining virtual care hinges upon transformative actions (rather than adaptive actions) that strengthen health systems so that they can face the dynamic and emergent challenges associated with COVID-19 and other shocks. Based on the barriers and facilitators we identified, we present the lessons we learned and recommend transformations for sustaining virtual care during and beyond the COVID-19 pandemic.

## Introduction

### Virtual Care Adoption During the COVID-19 Pandemic

Virtual care has long faced a perplexing paradox; despite having enormous promise, the incidence of widespread adoption has remained sparse [1]. However, amid the global COVID-19 pandemic, health systems have rapidly adopted and expanded virtual care technologies at an unprecedented scale and pace [2,3]. Virtual care refers to “any interaction occurring remotely between patients and/or members of their circle of care, through any form of communication or information technology with the aim of facilitating or maximizing the quality and effectiveness of patient care” [4]. Such interactions may be synchronous or asynchronous and can be mediated through a variety of technologies, including video consultations, telemonitoring, and electronic medical records (EMRs). These technologies have played a pivotal role in facilitating access to health care during the pandemic [5], especially for patients with chronic illnesses who are at higher risk of severe illness from COVID-19 [6,7]. As nations plan for the provision of future health care services, what remains in question is: how can rapidly virtualized health care services be effectively sustained?

### Health Systems Resilience

Inherent to both the response to COVID-19 and virtual care adoption is complexity, in that both are fraught with nonlinearity, unpredictability, and interdependencies [8,9]. In the face of acute or chronic [10,11] stressors or challenges (also known as “shocks”) [12], an imperative for health systems is to be resilient [13]. Health systems resilience is commonly characterized as “the capacity of health actors, institutions, and populations to prepare for and effectively respond to crises; maintain core functions when a crisis hits; and, informed by lessons that are learned during the crisis, reorganize if conditions require it” [14]. Of importance is not only the ability to return to equilibrium after experiencing shocks but also the ability to create a new equilibrium, especially when shocks are persistent and intense. Blanchet et al [15] describe resilience processes as absorptive, adaptive, and transformative. With suitable preparation, health systems may absorb some shocks without considerable changes in the amount or allocation of resources. Greater demands however require systems to adapt policies and workflows and reallocate resources. As demands on the system increase in intensity or duration, systems may need to transform by fundamentally changing the services or procedures they offer.

As the global COVID-19 pandemic shifts from an acute shock to a chronic shock, health systems will need to demonstrate continued resilience. With the need to deliver health care remotely during the COVID-19 pandemic, adopting and sustaining virtual care may constitute important components of health system resilience. Although virtual care adoption has been a prominent subject of research [16-19] that has largely been enabled by theories such as the Unified Theory of Acceptance and Use of Technology (UTAUT) [20], such theories often neglect the broader context in which virtual care adoption occurs [21,22], thereby limiting their ability to capture the novel phenomenon of virtual care adoption during the COVID-19 pandemic. Therefore, it is equally important to align evaluations of virtual care adoption with health system priorities, such as resilience, to complement the existing literature on virtual care adoption. Yet, few studies emerging from the evolving literature on COVID-19 have discussed virtualization efforts from this perspective.

To facilitate learning from the initial phase of the pandemic [13], the objective of this paper was to report patient, clinician, and staff experiences with the virtualization of cardiac care, and the perceived sustainability of the virtual care model during and after the pandemic. The research question was as follows: what were the experiences, barriers, and facilitators related to the rapid virtualization of cardiac care during the COVID-19 pandemic?

## Methods

### Setting

The Peter Munk Cardiac Centre Heart Function Clinic at Toronto General Hospital in Ontario, Canada began a marked expansion of virtual care delivery on March 9, 2020. This occurred 2 days before the World Health Organization announced the COVID-19 global pandemic [23]. Between April and September 2020, 1113 scheduled in-person visits were converted to virtual visits by the Ontario Telemedicine Network (n=134, 12%) or by phone (n=979, 88%). Clinicians affiliated with the clinic also had remote access to the hospital’s EMRs, which centralizes documentation and decisions related to the patient’s care. Clinicians also had the option to enroll patients in the Medly program—a mobile phone–based telemonitoring program for patients with heart failure. The program uses a rules-based algorithm [24] that delivers tailored self-care messages to patients and clinical decision support based on the daily input of weight, blood pressure, heart rate, and symptom data. The program, which is described elsewhere [25], was designed to support patients’ self-management and promptly identify symptom deterioration between regularly scheduled in-person visits. The program became part of standard care in 2016. Clinical alerts were largely managed by a Medly coordinator (a registered nurse within the Heart Function Clinic), and alerts were escalated to cardiologists as required. To support the clinic’s rapid virtualization, two nurses from another cardiology department within the hospital were assigned to the Medly team on a part-time and temporary basis. No limits were established for the duration of enrollment in the Medly program. Most patients in the Medly program use their existing devices (phone, weight scale, and blood pressure monitor); however, equipment is made available to patients who do not have the means to supply their own devices.

### Study Design

This study used a single-case study design, with the case defined as the Heart Function Clinic [26]. In total, 3 embedded units of analysis—the use of virtual care by patients, clinicians, and operational staff—were selected to understand people’s experiences with and the barriers and facilitators of the rapid virtualization of cardiac care. This was a qualitative study that focused on semistructured interviews with the three participant groups.

### Recruitment of Participants

Patients, clinicians, and operational staff were recruited as part of an existing quality improvement study of the Medly telemonitoring program (University Health Network Research Ethics Board 16-5789 and University of Toronto Research Ethics Board 39449). All 12 clinical staff and 4 operational staff members from the telemonitoring program were invited to participate. Potential patient participants were identified based on demographic characteristics collected from a self-report questionnaire that was used for the Medly quality improvement study. Efforts were made to recruit participants across a range of demographic characteristics, including age, sex, the location of residence (urban, suburban, or rural), ethnicity, income, and comfort with technology. Eligible patients were those enrolled in the Medly telemonitoring program who could speak English.

### Data Collection and Analysis

Interview guides consisting of semistructured, open-ended questions were developed based on the Benefits Evaluation Framework [27]. Separate interview guides were developed and tailored to patients, clinicians (nurses and cardiologists), and operational staff. To accommodate physical distancing measures, in-depth, semistructured interviews were conducted over the phone by two authors experienced in qualitative research (AS and SW). Phone interviews were conducted between May 4, 2020, and June 18, 2020, and lasted approximately 30 minutes. Participants were asked to comment on their experiences with managing heart failure as well as their experiences with using virtual care technologies during the COVID-19 pandemic (including but not limited to virtual consultations and telemonitoring). All interviews were digitally recorded and professionally transcribed verbatim for analysis.

An inductive thematic analysis was conducted at the semantic level according to the iterative, 6-phase approach outlined by Braun and Clarke [28]. Three authors were involved in the data analysis process (MG, AS, and SW). To improve the trustworthiness of the analysis, all authors engaged in both procedural and analytical memoing throughout the research process [29]. Transcripts and analytic memos were entered into NVivo 12 (QSR International) [30] which was used as an organizational tool to collate the data and facilitate coding (eg, creating, sorting, reordering, and merging codes). One author (MG) independently analyzed all interview transcripts to gain a holistic perspective on all of the collected data. In parallel, two authors independently analyzed either patient (AS) or clinician and staff (SW) transcripts. Authors initially met to compare and discuss codes for each participant group. At this stage, codes were clustered into categories to identify predominant themes for each participant group. After a series of 4 analytic discussions, the research team collectively developed 5 themes. The final set of themes was reviewed for internal coherence, consistency, and distinctiveness by the wider research team [28,31].

## Results

### Participants’ Characteristics

A total of 29 participants were interviewed, including 16 patients, 5 cardiologists, 4 Medly nurse coordinators (including new, temporary nurses), and 4 operational staff members. The characteristics of interviewed patients are presented in Table 1.

**Table 1:** Characteristics of patient interview participants (N=16).

Characteristic		Value
Age (years), mean (SD; range)		54.5 (SD 19.9; 23-78)
**Sex, n**		
	Male	8
	Female	8
**Ethnicity, n**		
	White	10
	Black	1
	Filipino	1
	South Asian	1
	Southeast Asian	2
	Not declared	1
**Place of birth, n**		
	Canada	12
	Other	3
	Not declared	1
**Highest education achieved, n**		
	High school	2
	Trade or technical training	4
	College or university	8
	Postgraduate	1
	Not declared	1
**Rurality, n**		
	Urban	4
	Suburban	8
	Rural	3
	Not declared	1
**Living arrangement,**		
	Living with family or partner	13
	Living alone	2
	Not declared	1
**Income (CAN $ [US $]), n**		
	<15,000 (<11,998.80)	4
	15,000-49,999 (11,998.80-39,995.30)	3
	50,000-74,999 (39,996.10-59,993.40)	6
	>75,000 (<59,994.20)	1
	Not declared	2
**Comfort with technology, n**		
	Very comfortable	3
	Somewhat comfortable	2
	Comfortable	4
	Not comfortable	2
	Not declared	5

### Interview Findings

The following five themes were identified in the analysis of interview data: (1) patient safety as a catalyst for virtual care adoption; (2) piecemeal virtual care solutions; (3) confronting new roles and workloads; (4) missing pieces in virtual care; and (5) the inequity paradox.

#### Patient Safety as a Catalyst for Virtual Care Adoption

As fears related to COVID-19 heightened and widespread physical distancing measures were established, patients and clinicians questioned the safety of the hospital environment. Patients and clinicians were acutely aware that individuals with pre-existing conditions were at an increased risk of developing a severe illness resulting from COVID-19 contraction. Maintaining patient safety through hospital avoidance was thus a key motivation for patients and clinicians to reassess the role of virtual care in heart failure management. Virtual care was no longer seen as an option for complementing in-person care but rather as the sole care option for many patients in nonurgent circumstances. For example, a patient said:

...[going to the hospital] could be a little bit worse knowing my situation and maybe I could get close to someone and get this COVID, and maybe it could even be the opposite. So that’s why as much as I’m [wanting] to see the doctor, I wanted to stay away also.

Patient 1

A clinician also stated:

…in large part, because we don’t want patients unnecessarily exposed to potential COVID, we have moved to a virtual care environment to improve the safety of patients.

Clinician 9

When newly adopting virtual care technologies or expanding their use of virtual care, patients and clinicians weighed the perceived benefits of virtual care against its burden. For many, maintaining patient safety by facilitating hospital avoidance presented a new benefit to virtual care that outweighed previous reservations. For example, enrolling patients in the Medly program comforted clinicians when postponing clinic visits for stable patients, as they knew that symptom deterioration would be identified early. This was done to increase clinician capacity and ensure that their attention could be focused on treating the most at-risk patients and planning service restructuring processes at the peak of the pandemic’s first wave. One clinician stated:

“...with the volume of patients that we’re now seeing virtually—right at the beginning it was very helpful to onboard some of my sickest patients and then I knew at least they were being tracked by [telemonitoring].”

Clinician 8

Although new benefits to virtual care emerged during the COVID-19 pandemic, these did not sufficiently outweigh the burden for a small minority of interviewed patients. For these patients, the personal benefits of virtual care were unclear and thus did not justify the new work involved, even when the monetary costs of participation were covered by the health care system (ie, equipment provided by the program). For example, a patient stated:

...it doesn't cost me anything...but it just is not beneficial to keep doing [telemonitoring]…I'm not the type of person that wants to measure everything—check my weight, and check this, and check that every single day. You know it diminishes the quality of life if you have to subject yourself to this sort of regimentation.

Patient 2

#### Piecemeal Virtual Care Solutions

To accommodate physical distancing restrictions and the need to work from home, clinic appointments were cancelled, deferred to a later date, or changed to virtual visit appointments. Multiple virtual care technologies, including existing and new technologies, dedicated technologies (eg, EMRs and telemonitoring systems), and general-purpose technologies (eg, phone calls and FaceTime; implemented after obtaining consent), were rapidly deployed using a piecemeal approach to facilitate virtual visits. A clinician said:

...we’d had a good experience of [telemonitoring] already, so it was kind of a no-brainer to try and onboard as many patients as would be appropriate to the [telemonitoring] platform, and follow them that way, in conjunction with the telephone follow-ups or Ontario Telehealth visits to try and keep them physically out of the hospital.

Clinician 2

The adoption of multiple virtual care technologies by clinicians allowed many patients to newly engage with or expand their use of virtualized care. Using multiple virtual care technologies to connect with the health care system was perceived as positive by patients, as they thought that using such technologies would help them overcome the limitations of each virtual care technology. For instance, data collected through the Medly system, which was originally designed to provide care between in-person appointments, were also used to provide additional context for virtual visits and allow for safe and effective remote medication titration. With different types of information captured and provided by various technologies, patients felt reassured that the quality of their care was maintained despite the reduced capacity of the health care system to see them in-person. One patient stated:

…it's weird because the doctor can't see me, right?...But my first appointment I didn’t have a scale and I didn’t have the blood pressure—it was pre-[telemonitoring]…it's definitely comforting to know that the [telemonitoring] program does exist.

Patient 3

For clinicians however, the value of using multiple virtual care technologies was mixed. A piecemeal approach to virtual care allowed clinicians to act rapidly, as it provided the flexibility needed to select technologies based on their needs and backup options when technical challenges occurred (eg, switching to a phone call when a video call freezes). Yet, switching between multiple siloed virtual care systems often duplicated administrative work that reduced care efficiency. To improve the sustainability of virtualized clinic services, clinicians expressed a strong desire for connectivity between virtual care systems:

It is extremely important, I think, having [telemonitoring] connect with our online EMR system, so it does pull the blood work, but it doesn’t pull other things. We have to manually input medications, which is very tricky...Everything in one system would allow us to work a lot more seamlessly and it would be more efficient, and it would be possible probably to look after more patients if everything was combined.

Clinician 4

#### Confronting New Roles and Workloads

As workspaces shifted from clinic to home, clinicians had to learn to work with reduced administrative capacity. Working efficiently from home without timely and convenient access to administrative, clinical, and lab systems posed a challenge to clinicians and staff. For instance, a clinician said:

I'm a little bit more preoccupied about not having the paper trail and that things are going to fall through the cracks. We had very robust mechanisms in place to sort of make sure that things weren't missed and I'm a little bit more worried about that happening with virtual care.

Clinician 6

The reduced support when working from home, lack of clarity regarding transitioning roles, and compromised administrative safety net during clinic virtualization meant that clinician workloads unexpectedly increased with the addition of extra tasks in an ad hoc manner. As more patients were onboarded to the Medly program amid the pandemic, the program faced unique challenges to scaling up its operations and delivery methods. One staff member said:

...while we were also addressing this quick ramp up, we were also figuring out our roles in terms of how we would split up that person’s responsibilities among the numbers who were left.

Staff 4

A clinician also stated:

The numbers of patients that I'm contacting on the phone are fewer than the patients we would see in the clinic. The reason for that is that the phone follow-ups and documentation and paperwork take longer. It’s more cumbersome than if we were physically on-site at the clinic. The other reason is that – we’re just one person.

Clinician 2

Concurrent with the added administrative duties, clinicians also faced changed dynamics with patients. With virtual care, the onus was on clinicians to reach out to patients at home instead of on patients, who were previously expected to meet clinicians at the clinic. Thus, barriers to the clinical encounter that were traditionally experienced by patients (eg, delays, waiting times), were now experienced by the clinicians, thereby generating new frustrations. One clinician said:

Trying to find patients is a little bit more difficult than patients trying to find us. What I mean by that is that there’s a lot of time that is wasted in chasing patients down when they don't pick up the phone.

Clinician 6

Changes to roles and patient-provider dynamics sometimes resulted in clinicians feeling less satisfied with their job when working remotely. This negative impact on their job satisfaction impacted their perceptions of virtual visits as a sustainable option. Another clinician stated:

I think most physicians didn’t sign up to make 50 phone calls a day. None of us trained to [be] sort of...telemarketers. It’s kind of what you feel like, right? Making call, after call...It’s not that much fun. Now clinic is clinic, but it’s the interaction with the patients in person that kind of like make it worth it and I don’t think any of us really signed up for this.

Clinician 8

#### Missing Pieces in Virtual Care

Patients and clinicians expressed the need to make virtual care interactions more clinically and personally meaningful. Structured information collection via certain virtual care technologies was thought to limit the type of information patients could communicate to their health care teams. Moreover, routine diagnostic exams took longer to complete during the pandemic, which further delayed decisions about patients’ care. Visual assessment, touch, and diagnostic exams were some of the elements missing in virtual care that hampered a comprehensive and timely assessment. For example, one clinician stated:

You miss the physical examination to see the patient, like the things that we do with our eyes. Because there are some patients that complain about everything and there are some patients that don't say anything. So those two cases are very difficult to assess if you don't have objective assessment...We have [objective assessment] with a delay, which is annoying.

Clinician 5

Patients and clinicians also had fewer opportunities to interact directly with each other in this new setting. For example, clinicians mentioned that they spoke with patients’ caregivers (eg, family member) instead of patients. Patients who participated in the telemonitoring program would only be contacted by the health care team if they reported worsening or severe symptoms. Consequently, stable patients who only presented mild heart failure symptoms were less satisfied with their relationship with the health care team because they did not know how the program was impacting their care management. The following are statements from a clinician and patient:

Many times, we talk to one person, whereas in clinic, usually if the patient comes with someone else, we'll talk to both...I always like to interact with my patients directly and you miss that with virtual care.

Clinician 5

But to me, it’s just stated that I’m feeding [information] automatically to some black hole. And I don’t know what’s coming out of it or what will ever come out of it except if they go out of the parameters.

Patient 4

One clinician reflected upon how the COVID-19 pandemic changed their perceptions of their previous experiences with implementing virtual care at another clinic. From the COVID-19 pandemic, they learned that asking about how virtual care could overcome the limitations of in-person care was more useful than comparing virtual care to in-person visits. They said:

[The clinicians at the other clinic] didn’t even ask the patients; they asked the doctors. “Do you think the video was as good as in-person?” And they said no. and so we said “OK, we’re going to scrap this approach.” In my opinion that was the wrong question to ask because, of course, in person is better. But the question was “[is] this better than not any visits? And was it adequate?” And the answer would’ve been certainly yes.

Clinician 8

#### The Inequity Paradox

It was widely accepted among participants that virtual care technologies were integral for facilitating access to cardiac care during the COVID-19 pandemic. However, clinicians had different views on how these technologies would impact people’s access to care after the pandemic. For example, one clinician said:

I don’t foresee clinics going back to the way they were. I think they’ll be reserved for people who are unwell or who need their diagnostics done.

Clinician 3

Another stated:

The ones that can afford it, the ones that want to see their doctor, they’re going to want to come see their doctor again even if they could do that virtually. But for [some of] the patients...the risk/benefit ratio really favors just sitting and doing it from home.

Clinician 8

A critical barrier of sustaining virtual care was its paradoxical impact on inequities; while virtual care technologies could potentially improve the distribution of health care services, they often targeted patients who already had access to health care. Thus, as populations with access to care enjoyed faster and more convenient care, inequities continued to widen. One clinician said:

I’ll give you examples of patients that are the highest risk patients—and I see a lot of patients that were recently admitted—but you take the homeless people, the people that are under-housed with a touch of dementia…Like [telemonitoring] is not going to work for them. And those are exactly who you need it to work for.

Clinician 8

Clinicians rejected the notion that a single virtual care technology could serve the needs of all patients. Instead, a dynamic approach to virtual care involving an ecosystem of technologies that are allocated based on the needs and means of patients was envisioned for the future. A clinician stated

…not losing humanism and not losing the patient perspective about what things should or shouldn’t be pushed versus pulled by [patients] is part of what we need to figure out as we move digital health forward…We’re still pushing things at patients; we haven’t been able to provide a venue of tools and an explanation of what those tools are.

Clinician 9

## Discussion

### Principal Findings

Scholars have argued that pandemics are opportune times for strengthening health systems [32]. Yet, few have explored the role of virtual care in health systems resilience amidst shocks, especially in high resource settings. In this study, we sought to understand the experiences, barriers, and facilitators of the rapid virtualization of cardiac care during the COVID-19 pandemic from the perspective of patients, clinicians, and staff. Across the five themes identified in this study, it was found that the motivation to protect patient safety and a piecemeal approach were factors that facilitated the rapid virtualization of cardiac care, whereas ad hoc virtual care roles and workflows, difficulties in building patient-clinician relationships, and widened inequities served as barriers. Through the lens of health systems resilience, we found that the large and likely prolonged disruption to the Heart Function Clinic that was introduced by the COVID-19 pandemic prompted resilience processes for maintaining cardiac care services. This study illustrates how virtual care can facilitate health systems resilience despite shocks that hinder or constrain health care delivery.

This study reveals that the adoption and expansion of virtual care within the Heart Function Clinic allowed absorptive (ie, new uses of existing virtual care technologies) and adaptive resilience (ie, the reduced number of in-person appointments) to mitigate the impacts of the COVID-19 pandemic. We observed that the COVID-19 pandemic created conditions in which the motivation to protect patient safety acted as an organizing vision that promoted the adoption and expanded use of virtual care technologies [33]. Drawing upon the UTAUT [20] may explain how these factors shaped clinicians’, patients’, and staff members’ behavioral intentions to use virtual care during the pandemic and in the future. According to the UTAUT, performance expectancy (ie, the benefits introduced to end-users after completing a task) shapes users’ behavioral intentions to use a technology [20]. Our research shows that the conditions associated with the pandemic changed the performance expectancy of virtual care by promoting its increased adoption. In particular, virtual care was perceived to have a greater relative advantage within a pandemic context, as patients and clinicians sought to avoid nonessential, in-person hospital visits. Findings that have been corroborated elsewhere have shown that reduced rates of emergency department visits and hospitalizations for heart failure were observed during the early phase of the pandemic [34]. As circumstances evolve with the COVID-19 pandemic, patient and clinician interest in, and use of, virtual care may shift as in-person settings are perceived to be safer. Consequently, the relative advantage [20] of virtual care may decrease as circumstances improve. Continuing to frame virtual care as a safety net for traditional, in-person care (regardless of whether in-person delivery has been restricted) may facilitate its sustained use by patients and clinicians.

A piecemeal approach that involved using dedicated and general-purpose technologies was critical for providing a rapid response. However, this approach must always follow organizational and jurisdictional policies about patient privacy, such as the need for patient consent and compliance with the Personal Health Information Protection Act. The use of general-purpose tools within clinical care might reflect the fact that robust telehealth tools were not yet available in settings that did not have existing virtual care options for absorbing shocks to in-person delivery. Alternatively, this may reflect the unanticipated technical challenges (eg, poor quality and dropped calls) that clinicians faced when using dedicated technologies. Other studies have documented the widespread adoption of general-purpose videoconferencing tools, such as FaceTime, Skype, and Zoom, during the COVID-19 pandemic [35]. Although the use of these off-the-shelf technologies allowed the Heart Function Clinic to act rapidly, our findings suggest that it inadvertently introduced or duplicated tasks that hindered clinician efficiency. Tailored virtual workflows for bridging multiple platforms were strongly desired by clinicians in order to work in a virtual care environment.

While the rapid virtualization efforts instated by health care settings are to be celebrated, we argue that they remain fragile to the prolonged and intense nature of COVID-19 and future shocks placed on health systems. Long-term reliance on adaptations to the pandemic, which Lee et al [36] called “coping,” will likely prove to be insufficient without appropriate transformations to roles, clinical workflows, and infrastructures. Indeed, in this study, the adaptations to cardiac care were perceived as inadequate for sustaining virtualized clinic services. The drastic loss of administrative infrastructure when working in a virtual care environment led to perceptions of reduced productivity and increased workloads from clinicians. Similar impacts on clinician productivity have been well documented [37], and emerging studies have reported a considerable decline in the overall number of appointments during the pandemic despite the provision of virtualized clinical services (eg, a decrease of 25%) [35]. Revisiting clinic roles and designing workflows that are tailored to virtual care were desired by the interviewed clinicians and staff.

Workflow challenges were compounded by the limited types of data that could be captured by virtual care technologies. This made the development of meaningful patient-clinician interactions difficult. Patients in this study perceived relationship quality based on the frequency and content of the feedback (both automatic and on-demand feedback) they received from virtual care technologies. When feedback fell short of their expectations, patients’ perceptions of virtual care were negatively impacted. We posit that unclear expectations for virtual care may stem from the fact that dedicated virtual care technologies deployed during the COVID-19 pandemic were designed and implemented to fulfill purposes that were different from their roles in the COVID-19 pandemic. For example, virtual visits were previously considered as a care option; however, they are now regarded as essential during the pandemic. As many virtual care technologies are being used in expanded ways (eg, replacing care visits instead of complimenting them), adaptations to existing virtual care technologies are needed so that they can continue to operate within this new context.

Although we observed that virtual care provided the patients of Heart Function Clinic with an essential health care service during the pandemic, only a small portion of patients could participate in virtual care. Clinicians in this study reported that the barriers to virtual care and in-person care were largely the same. However, improving the convenience and speed of care delivery for those who could access virtual care resulted in widened inequities. As similar findings about the digital divide have emerged during the pandemic [38], characterization of, and adaptations for, various underserved groups are essential for preventing the further widening of gaps.

Leadership and governance have been identified as critical components of health systems resilience [13,39]. It is thus important to note that this study occurred within a context of strong governance and quality improvement leadership. Strong leadership not only enabled resilience capacities for clinical purposes but also allowed for the rapid evaluation of interventions. Health systems facing similar shocks may benefit from facilitating similar leadership commitments to research and quality improvement during the COVID-19 pandemic. Such actions will facilitate the oft-forgotten component of learning that is integral to continued health systems resilience [13]. Our rapid evaluation serves as an indicator of learning from the early stages of the COVID-19 pandemic, and it will continue to guide efforts throughout and beyond the pandemic. A critical issue that remains with respect to governance is identifying the leadership capacities that are needed to facilitate transformations in health care settings that promote virtual care sustainment. Transition management principles [40], which are used to “explore, understand, operationalize, guide and accelerate transitions with networks of change agents” [41], can offer guidance. This approach to planning and governance can not only benefit the transformation of health care and promote virtual care sustainment, but also prepare health systems for future shocks [41].

### Recommendations

Our research highlights opportunities for transformative resilience, which, if realized, will assist in the sustainment of virtualized clinic services throughout and beyond the pandemic. In light of the study findings, we offer recommendations to promote virtual care sustainment (Textbox 1).

**Recommendations for promoting virtual care sustainment.**

**Recommendation 1: Invest in a virtual care ecosystem that acts as a safety net for in-person care**

- Curate resources and technologies for virtual care that will support clinical management from afar.
- Design context-based and patient-specific recommendations for patients who experience worsening symptoms.

**Recommendation 2: Streamline tasks that rely on multiple technologies**

- Minimize interruptions from multitasking and enable the cross-publishing of information across virtual care technologies.
- Backup options should be established to limit the impact of technical issues (eg, allow clinicians to immediately switch platforms as needed).

**Recommendation 3: Redesign roles and workflows to support collaboration**

- Consult with clinicians, staff, and patients to devise innovative workflows that take advantage of task sharing to increase care provider efficiency.
- Maintain some level of redundancy between roles and tasks (eg, cosharing responsibilities for patient education, education, and follow-ups) to reduce the impact of single points of failure in a virtual workflow.

**Recommendation 4: Personalize follow-up systems to achieve the desired intensity of care**

- Consult with patients and clinicians to identify their preferences in terms of the mode (eg, video, voice, or text), frequency (eg, the amount of times a patient should be contacted by the health care team), and delivery (eg, synchronous or asynchronous delivery) of messages among the health care team.

**Recommendation 5: Revisit patient groups served by virtual care**

- Characterize the population served by the clinic in terms of age, ethnicity, gender, and geographical location to identify potentially underserved groups.
- Revisit affordability, usability, and availability requirements to ensure that patients in communities without high-speed internet connections can have access to virtual care [42].

### Limitations

There are several limitations to note. First, as all patient participants were enrollees of a telemonitoring program, our findings may not reflect the views of individuals who solely attended video and phone visits. Second, due to physical distancing measures, in-person interviews were not possible at time of data collection. As such, phone-based recruitment and data collection may have resulted in a greater representation of patients who feel comfortable with technology. Third, although health systems resilience is a global health priority, this study was conducted in a high-resource setting. As resilience capacities may differ in low-resource settings, the role of virtual care in these contexts warrants further exploration. Fourth, three cardiologists from the Heart Function Clinic were not represented due to scheduling challenges. Finally, despite our efforts to purposefully recruit participants with a range of demographic characteristics, the patients we interviewed were predominately young, White, residing in suburban areas, and college educated. Although we believe that our sample was representative of the patient population of the Heart Function Clinic, our sample is unlikely to be reflective of the broader population with heart failure in terms of age, ethnicity, rurality, and education. As such, our study may have potentially overestimated patients’ use of and experiences with virtual care. Further research with more diverse samples is needed.

### Conclusions

As health systems face shocks such as the global COVID-19 pandemic, virtual care technologies have been critical enablers of health systems resilience. In this study, we report that the adoption and expansion of virtual care enabled absorptive and adaptive resilience of cardiac care. This transition was largely motivated by a need to maintain patient safety and facilitated by a piecemeal approach to virtual care adoption. Despite the absorptive and adaptive resilience demonstrated by cardiac care services, we identified barriers that were experienced by patients, clinicians, and staff within a virtual care environment, including a lack of administrative support, the use of ad hoc virtual care roles and workflows, difficulties in building patient-clinician relationships, and widened inequities. If left unaddressed, these barriers threaten the sustainment of virtual care, thereby leaving the opportunity to strengthen health systems through virtual care unrealized. We argue that resilience processes that are implemented during the COVID-19 pandemic need to be transformative. This involves the reconsideration of clinical roles and workflows, the redesign of virtual care systems, and active efforts for engaging populations that continue to be underserved. To assist health settings, we present recommendations for promoting virtual care sustainment, which will help them build resilience to the shocks inherent in and created by complex processes within complex adaptive systems, such as the health care system. Through such transformations, health systems enduring shocks may emerge strengthened and more resilient than before.

## Abbreviations

EMR: electronic medical records
UTAUT: Unified Theory of Acceptance and Use of Technology
